# Transcriptomic analysis reveal an efficient osmoregulatory system in Siberian sturgeon *Acipenser baeri* in response to salinity stress

**DOI:** 10.1038/s41598-018-32771-x

**Published:** 2018-09-25

**Authors:** Baoying Guo, Zurong Tang, Changwen Wu, Kaida Xu, Pengzhi Qi

**Affiliations:** 1 National Engineering Research Center of Marine Facilities Aquaculture, Marine Science and Technology College, Zhejiang Ocean University, Zhoushan, 316004 China; 2 Key Laboratory of Sustainable Utilization of Technology Research, Marine Fisheries Research Institute of Zhejiang, Zhejiang Zhoushan, 316021 China

## Abstract

Sturgeons are euryhaline fish species that have developed specific mechanisms of osmotic and ion regulation to adapt to waters of varying salinity. For the aim to elucidate the osmoregulation strategy behind its high salinity tolerance of sturgeons, the transcriptomes of gills in Siberian sturgeon *Acipenser baeri* under salinity stress (30 ppt) were sequenced using deep-sequencing platform Illumina/HiSeq-2500 and differential expression genes (DEGs) were identified. A total of 167, 501, 278 clean reads were obtained and 280, 238 unigenes were composed of those clean reads with the mean length of 520nt, and the N50 of 630 bp. Unigenes Sequence alignment was implemented via KEGG, KOG, NT, NR, PFAM, Swiss-Prot, and GO databases. 62, 242 unigenes (22.21%) were annoated in at least one database. 11380 significantly differentially expressed unigenes were found, 6969 of which were up-regulated and 4411 were down-regulated by salinity stress. Amongst the top 20 KEGG pathways with the most amount of annotation sequences, some pathways such as glycerophospholipid metabolism, fatty-acid biosynthesis, glycolysis/gluconeogenesis, oxidative phosphorylation have been comprehensively proved to be relevant to osmoregulation. Despite of these, three possible osmoregulation-related signaling pathways as lipid metabolism related pathways, tight junction pathway and thyroid hormone signaling pathway have been widely analyzed in the current study. In all DEGs, some of the typical genes involved in osmoregulation, including calcium-transporting ATPase 4 (ATP2B4), Na^+^/K^+^-ATPase alpha subunit (α-NKA), potassium-transporting ATPase alpha chain 1 (ATP4A) and Ras GTPase-activating protein (RasGAP) etc were also identified. RNA-seq results were validated with quantitative real-time PCR (qPCR), the 12 selected genes showed a consistent direction in both DGE library and qPCR analysis, proving that the RNA-seq results are reliable. The present results would be helpful to elucidate the osmoregulation mechanism of aquatic animals adapting to salinity challenge.

## Introduction

Sturgeons, generally refer to the fish species of family Acipenseridae, order Acipenseriformes. All the sturgeon species are chondrostean, which represent one of the oldest and rarest fish in the world. While they possess primitive characters, their physiological plasticity is great, which has determined their wide adaptation to varying environmental conditions^1^. Most of the sturgeons inhabit water bodies of varying salinity presently, despite of their origin as freshwater fish^2^. Moreover, the majority of sturgeons are anadromous fishes migrating between freshwater (FW) and seawater (SW) during their life cycle^3^. It is believed that sturgeons have developed specific mechanisms of osmotic and ion regulation to adapt to waters of varying salinity. However, the information attribute to osmoregulation to salinity stress in sturgeons is vacant to date. The elucidation of mechanism behind the adaption to the salinity fluctuations of sturgeons should contribute to the development of sturgeon culture.

Salinity has significant effects on physiology of aquatic organisms, and salinity adaptation is a complicated process that involves a series of physiological responses to the environment with different osmotic regulation requirements^4,5^. Euryhaline teleosts have a good adaptability to a wide range of ambient salinities. However, it is not clear about the osmotic regulation mechanism and the molecular response of these animals to salinity stress^6^. A set of structures such as the gills, kidney, and gut play a vital role in the regulation of osmoregulation of teleosts^7,8^. The gill is exposed to external environment directly entails great osmotic challenge of salinity. Gill contains complex transport epithelia functions as aquatic gas exchange, acid-base regulation and excretion of nitrogenous wastes^9^. Several studies have shown that euryhaline fishes reorganize the epithelium of gill considerably to meet the requirements of ion transport and permeability^10,11^.

In recent years, high-throughput sequencing technology has achieved great development and it has been widely used in many aquatic species. Compared with traditional technology such as cDNA microarray and suppression subtractive hybridization (SSH), high-throughput sequencing is fast and extremely sensitive, it can examine RNA transcriptions and acquire digital gene expression tag profilings without genomic reference sequences^12,13^. The analysis of transcriptomic profiling of species without whole-genome sequence information emerged as a forceful solution for genome study and identification of functional gene. It has made some progress in clarifying osmoregulatory mechanism under salinity stress. The analysis of osmoregulation-related 37 differentially expressed genes and 200 pathways were performed in the gills of *E. sinensis*^14^. Lv *et al*.^15^ identified that 552 differentially expressed genes including amino acid transport proteins and ion transport enzymes were obtained in *P. trituberculatus* under low salinity. Similarly, Hu *et al*.^6^ reported 585 genes differently expressed under low salinity from *L. vannamei* and 10 selected genes were validated with quantitative real-time PCR (qPCR) technique.

With their susceptible condition and economic significance in aquaculture, sturgeons are exposed to particular risks of population decline and extinction^16^. For the promotion and diversification of sturgeons farming, efforts are being made to implement such as breeding in salt water and seawater. Siberian sturgeon *Acipenser baeri*, mainly distributed in the siberian rivers between the Ob River and the Korema River in Russia, are extremely economical fish. In particular, caviar produced from its eggs is known internationally as “black gold” and “black pearl.” In recent years, this sturgeon species was successfully introduced to China and has achieved as one of the most important species for aquatic farming^17^. In this study, the transcriptome of *A. baeri* gills was detected by Illumina/Hiseq-2500 RNA-seq technology for the first time. The transcriptomic data of A. *baeri* between control group and salinity challenge group were compared and analyzed to identify osmoregulation-related genes and pathways. The discoveries of this study can be conductive to illustrate the mechanism of osmotic regulation for aquatic animals adapting to salinity challenges.

## Materials and Methods

### Ethics Statement

All experimental procedures were implemented in conformity with institutional guidelines, and protocols were approved by the Institutional Animal Care and Use Committee in Zhejiang Ocean University, Zhejiang, China.

### Animals

Siberian sturgeon juvenile individuals (55.0 ± 0.5 g in weight, 13.5 ± 1.4 cm in length) were purchased from Institute of Fisheries Research, Jiangsu Province, China, and these individuls were cultured in 12 m × 1.5 m (diameter × height) aquaria with a light-dark cycle of 14 h and 10 h at 26 °C for one week before treated. The water was half renewed every two days and commercial forage was feeded daily.

### Salinity challenge

When the experiment initiated, 60 fishes were randomly divided into control group and salinity challenge group, and each group consisted of three replicates, with 10 fishes in each replicate. These fish were transferred to 1200 L aquaria with the same culturing conditions during the test. The salinity of local naturally seawater is approximately 30 ppt, accordingly, 6 gradient salinity were chosen and each gradient was 5 ppt elevated. In the progress of salinity challenge, 600 L volume of each aquaria was used. When the water renewed, 100 L naturally seawater were supplemented (5 ppt elevated), and each salinity gradient was maintained for six days. During this period, the salinity challenge fish were transferred to clean water with the same salinity every two days.

The experiment was sustained for total 30 days until the challenge group was fully domesticated in naturally seawater with the ultimate salinity of 30 ppt. In the control group, sturgeons were always raised in freshwater. During the test, sturgeons of each replicate died no more than 30%.

### Sampling, RNA isolation, library construction and illumina sequencing

When the experiment terminated, three individuals from each duplicate were randomly sacrificed for tissue segregation, anaesthetized with a buffered solution of tricaine methanesulfonate (MS-222 100 mg/mL; Finquel, Redmond WA, USA) and immediately euthanized. The gills from three fish were pooled to reduce individual variation and immediately freezed in −80 °C until RNA extraction. In total, six mixed samples that were composed of three control samples and three challenged samples were eventually obtained for RNA extraction.

Total RNAs extraction were carried out with TRIZOL Kit (Invitorgen, Carlsbad, CA, USA) according to manufacturer’s proposals, and then treated by DNase I to implement the DNA digestion and obtain pure RNA products. And the employment of electrophoresis in 1.5% agarose gel was to check the integrity of RNA. NanoDrop 2000 Spectrophotometer (Thermo Scientific) at the absorbency of 260 nm was used to detect RNA quality and quantity.

An Illumina Tru-Seq™ RNA Sample Preparation Kit (Illumina, San Diego, CA, USA) was applied in generating sequencing libraries following the manufacturer’s protocol. After purifying, Illumina sequencing was carried out on an Illumina/Hiseq-2500 platform (Illumina, San Diego, CA, USA) to generate 100 bp paired-end reads by Novogene company in Beijing, China.

### De novo assembly and annotation

Filter of the raw reads were performed by taking out the primer sequence, low quality sequences and the adapters. Trinity software (v2012_10_05)^18^, was used to assemble the rest of clean reads. The Chrysalis clusters software was used to assemble and cluster the transcripts, and the longest one of each cluster was reserved and specified as “unigenes”.

Unigene annotation was performed with a sequence-based BLASTX alignment against nr, NCBI, COG, GO, KEGG, KOG, and Pfam database, both the E-value and the HMMER are less than 1.0e-5. The sequence direction of unigenes was identified by the best-aligning results.

### Differential gene expression analysis

The Bowtie 2 alignment tool (http://bowtie-bio.sourceforge.net/bowtie2) and RSEM method (RNA-Seq by Expectation-Maximization) were used to map all clean reads of two libraries to reference sequences for quantification^19^. The reference sequences were from assembled transcriptome data, and the number of readcount of each sample aligned to each gene was also determined. The FPKM method was applied to calculate unique gene expression levels^20^. Analysis of differential expression for sequence was performed with the data of readcount^21^. Significantly differential expression genes were selected based on Corrected P-value of 0.05 as the threshould. DESeq across the samples were further annotated via GO functional analysis and KEGG pathway analysis. All the differential expression genes (DEGs) were mapped to each term of GO database (http://www.geneontology.org/) and calculated gene number of each GO term.

### RNA-Seq data validation by quantitative real-time PCR (qPCR)

Twelve genes including Protein-tyrosine kinase 2-beta (PTK2B), Bone morphogenetic protein 3 (BMP3), E3 ubiquitin-protein ligase DTX4 (DTX4), Cytochrome c oxidase subunit 4 (COX4), Potassium-transporting ATPase alpha chain 1 (ATP4A), Cytosolic phospholipase A2 (CPLA2), Calcium-transporting ATPase 4 (ATP2B4), Na^+^/K^+^-ATPase alpha subunit (α-NKA), Potassium channel tetramerization domain containing 13 (KCTD13), Protein-tyrosine kinase 6 (PTK6), Heat shock 70 kDa protein (Hsp70) and Heat shock 90 kDa protein (Hsp90) were selected for confirmation of RNA-seq data by quantitative real-time PCR (qPCR) with a SYBR Premix Ex Taq kit (Takara, Dalian, China) in accordance with the manufacturer’s recommendations. The qPCR amplifications were conducted on ABI PRISM 7500 HT platform (Life Technologies, USA) and β-actin was set as the internal reference gene. Each sample was carried out in triplicate. The reactions were performed as follows: denaturation at 95 °C for 10 s, followed by 40 cycles of 95 °C for 10 s, and annealing for 30 s. At the end of qPCR assay, the melting curve was analyzed to evaluate the amplification specificity. The analysis of all data was implemented using the statistical software SPSS 17.0 (SPSS, Chicago, IL, USA) and presented as mean ± standard deviation (N = 3).

## Results and Discussion

### Sequencing and de novo assembly

Using high-throughput sequencing, 146, 905, 378 and 157, 041, 482 raw reads were obtained from gill tissue of control and salinity challenge Siberian sturgeons, respectively. After quality assessment, the control and challenge group yielded 143,101,118 and 153,400,160 clean reads with a Q30 percentage 91.80% and 92.07%, respectively. After alignment of these clean reads and the reference database, 89, 175, 420 (62.32%) and 97, 065, 408 (63.28%) were validated as the unique matches (Table 1). It was critical for reads to map unique sequences so that a transcript can be explicitly identified^22^. Regrettably, the transcriptomic research relevant to salinity adaption in teleost was rigidly scarce, and no literature was found to be conducted for the comparison of the reads mapped ratio. However, the transcriptomic analysis with salinity challenge has been realized in some aquatic invertebrate species, such as shrimp^6,23^, crab^14^, oyster^24^ and mussel^25^, and the mapped ratio was generally in accordance with our results, indicating that the high-throughput sequencing technology was efficient and reliable for the transcriptomic analysis of sturgeon gills. 389, 517 transcripts in total (mean length 629 bp) were predicted from the clean reads, and the N50 value was 947 bp. After removing redundancy, 280, 238 unigenes with the mean length of 520 bp and N50 value of 630 bp were assembled (Table 2).

**Table 1:** Summary of sequencing results.

Category	control group	Salinity challenge group
Raw reads	146, 905, 378	157, 041, 482
Clean reads	143, 101, 118	153, 400, 160
Q30 (%)	91.80	92.07
GC content (%)	44.55	45.89
Mapped clean reads	89, 175, 420	97, 065, 408
Mapped ratio (%)	62.32	63.28

**Table 2:** Length distributions of assembled transcripts and unigenes.

Length Range	Number of Transcripts (ratio, %)	Numbers of Unigenes (ratio, %)
300–500 bp	257, 439 (66.09%)	210, 640 (75.16%)
500–1000 bp	71, 540 (18.37%)	42, 391 (15.13%)
1000–2000 bp	40, 315 (10.35%)	17, 675 (6.31%)
>2000 bp	20, 223 (5.19%)	9, 532 (3.40%)
Total	389, 517	280, 238
Total length (bp)	245, 171, 366	145, 859, 444
N50 length (bp)	947	630
Mean length (bp)	629	520

### Annotation of functional classification

The entire functional annotations were shown in Table 3, after searching with the BLAST software of all unigenes against NR, NT, KO, KOG, Swiss-Prot, Pfam, GO and COG databases, 40, 225 (14.35%), 29, 184 (10.41%), 34, 688 (12.37%), 19, 963 (7.12%), 40, 056 (14.29%), 19, 055 (6.79%) and 40, 354 (14.39%) of all 280, 238 unigenes were found in individual database, respectively.

**Table 3:** Summary of annotations of assembled unigenes.

Annotated databases	Number of unigenes	Percentage (%)
Annotated in NR	40, 225	14.35
Annotated in NT	29, 184	10.41
Annotated in KO	19, 963	7.12
Annotated in Swiss-Prot	34, 688	12.37
Annotated in PFAM	40, 056	14.29
Annotated in GO	40, 354	14.39
Annotated in KOG	19, 055	6.79
Annotated in all databases	8, 720	3.11
Annotated in at least one Database	62, 242	22.21
Total unigenes	280, 238	100

**Figure 1:**
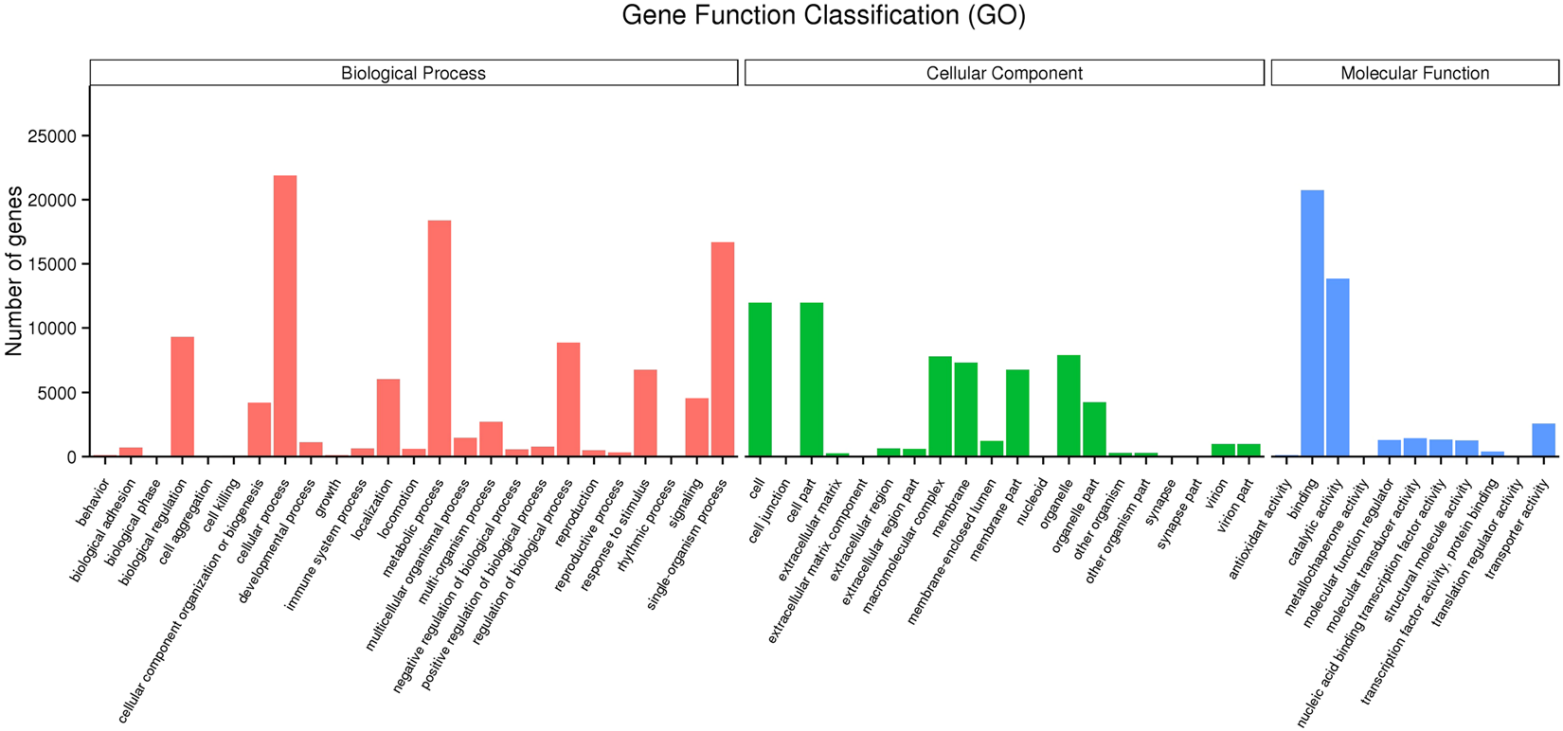
Gene Ontology (GO) classification of assembled unigenes. 40,354 unigenes in total were divided into three main categories: biological process, cellular component and molecular function. (For interpretation of the references to colour in this figure legend, the reader is referred to the web version of this article).

**Figure 2:**
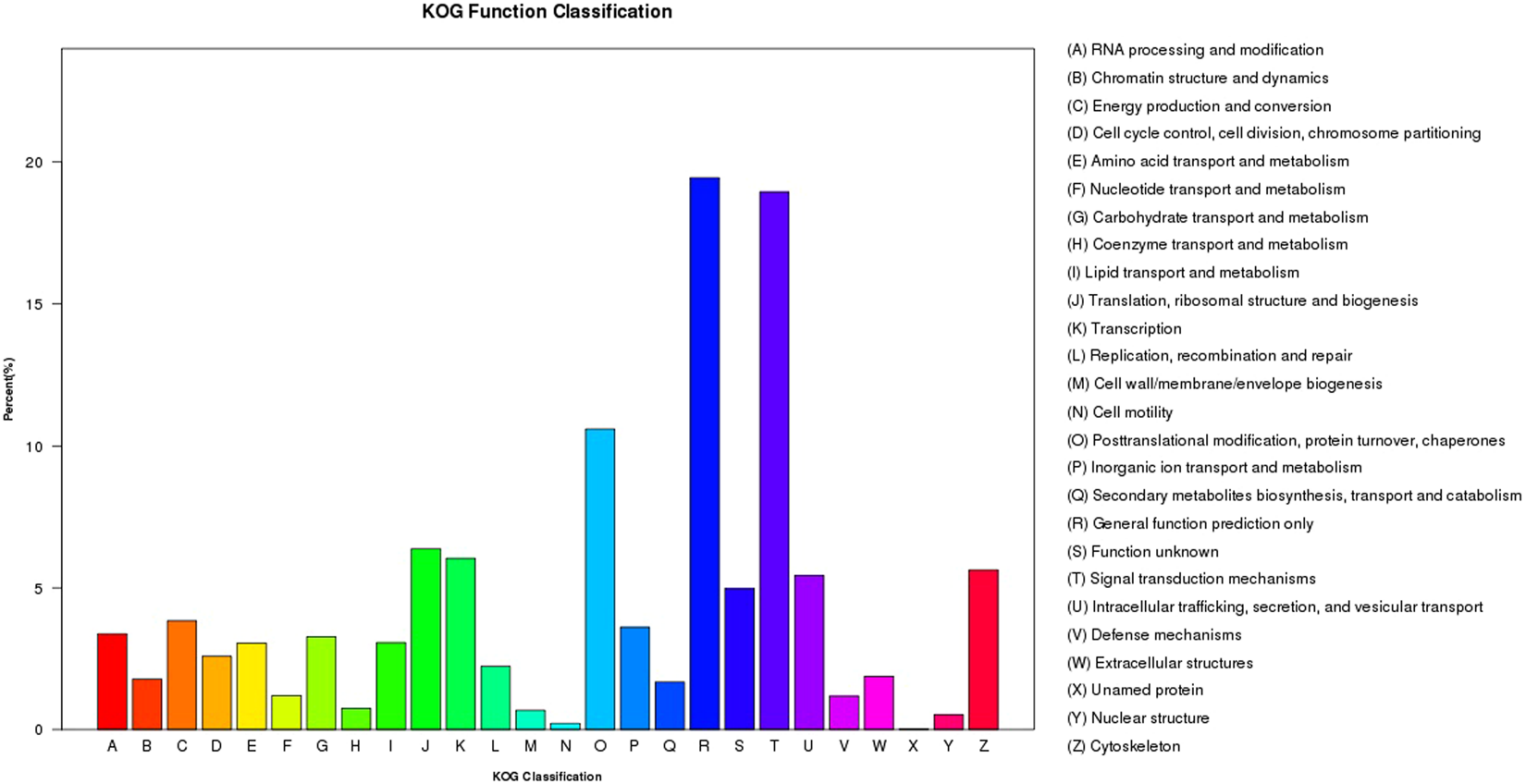
Eukaryotic Ortholog Groups (KOG) function classification of assembled unigenes. (For interpretation of the references to colour in this figure legend, the reader is referred to the web version of this article).

In this study, GO functional analysis indicated 40, 354 unigenes in total were classified into 56 sub-categories of three major categories: biological process, cellular component and molecular function. Among numerous biological process, the dominant group was cellular process (21, 892 unigenes), follwed by metabolic process (18, 388 unigenes) and single-organism process (16, 705 unigenes). For the category of cellular component, the dominant groups (11, 971 unigenes) were shared by cell part and cell sub-categories. As to the molecular function category, the most dominant groups were binding (20, 746 unigenes) and catalytic activity (13, 862 unigenes) (Fig. 1). Moreover, according to the results of the KOG annotation, 19, 055 unigenes in total were annotated and divided into 26 categories (Fig. 2), of which General Functional Prediction only (R) accounted for the the biggest proportion (3, 705 unigenes), Signal transduction mechanisms (T) held the second place (3, 611 unigenes), followed by post-translational modification, protein turnover, chaperon (O) (2, 020 unigenes).

### DEGs analysis

**Figure 3:**
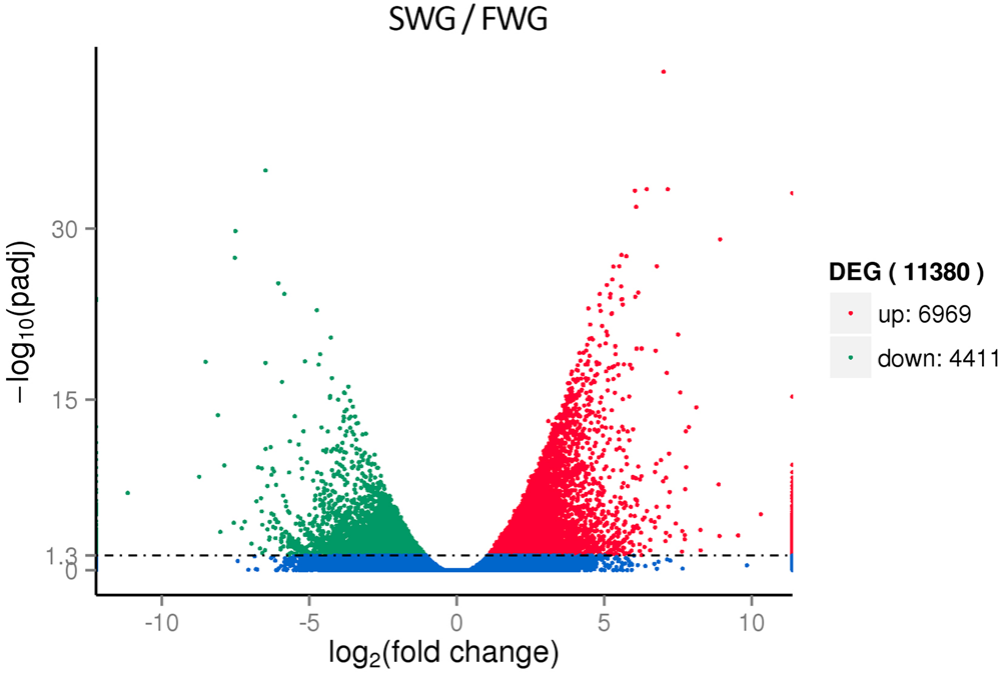
Volcano plot of differentially expressed genes identified between the salinity challenge group and control group. The green dots denote down-regulated gene expression, the red dots denote up-regulated gene expression, and the blue dots denote the gene expression without marked differences. (For interpretation of the references to colour in this figure legend, the reader is referred to the web version of this article).

In the process of differential expression analysis, FPKM was applied in calculating the relative unigene expression levels, and remarkably DEGs between two libraries were identified by a corrected padj <0.05 and log2 Fold Change (challenge group/control group) ≥1. After the salinity challenge, 11380 genes were differentially expressed, there were 6969 up-regulated genes and 4411 down-regulated genes (Fig. 3). Sturgeons are euryhaline fish which have migratory behavior between freshwater, estuarine and marine environments. During the migration from freshwater to seawater, sturgeons must drink water and excrete redundant ions actively through the gills to balance the loss of body water in the hyperosmotic environment. Salinity in the environment influences the morphology and ultrastructure of the gill epithelium^26–28^, which means, long-term alteration in the process of active transcellular ion transport of the gill epithelium happened during their acclimation. In the current transcriptomic analysis, a mass of genes were detected to differently expressed, which means numerous transcripts began to differentially modulated in response to salinity stress.

### Identification of DEGs related to osmoregulation

The top 20 KEGG pathways with the most number of annotated sequences were listed (Table 4). These pathways consisted of endocytosis, glycosaminoglycan biosynthesis, glycerophospholipid metabolism, Notch signaling pathway, steroid biosynthesis, fatty-acid biosynthesis, TNF signaling pathway, sphingolipid metabolism, phosphatidylinositol signaling system, tight junction, NF-kappa B signaling pathway, sphingolipid signaling pathway, oxidative phosphorylation, inositol phosphate metabolism, and etc. Amongst, some pathways as glycerophospholipid metabolism, fatty-acid biosynthesis, glycolysis/gluconeogenesis, oxidative phosphorylation have been comprehensively proved to relevant to osmoregulation. In the present study, three additional possible osmoregulation-related signaling pathways as lipid metabolism related pathways, tight junction pathway and thyroid hormone signaling pathway have been widely analyzed. In addition, in all DEGs, some typical genes related to osmoregulation, such as Calcium-transporting ATPase 4 (ATP2B4), Na^+^/K^+^-ATPase alpha subunit (α-NKA), Potassium-transporting ATPase alpha chain 1 (ATP4A) and Ras GTPase-activating protein (RasGAP) etc were also identified separately (Table 5).

**Table 4:** The top 20 KEGG pathways with the most number of annotated sequences.

KEGG ID	Pathways description	DEGs associated with the pathway	All genes associated with the pathway	P-value
ko04144	endocytosis	153	554	0.0011
ko00532	glycosaminoglycan biosynthesis	15	27	0.0012
ko00564	glycerophospholipid metabolism	42	127	0.0014
ko04330	Notch signaling pathway	45	142	0.0019
ko00100	steroid biosynthesis	14	26	0.0021
ko04350	fatty-acid biosynthesis	9	32	0.0024
ko04668	TNF signaling pathway	60	208	0.0025
ko00600	sphingolipid metabolism	23	58	0.0027
ko04070	phosphatidylinositol signaling system	60	210	0.0030
ko04530	tight junction	84	362	0.0061
ko04064	NF-kappa B signaling pathway	55	205	0.0119
ko04071	sphingolipid signaling pathway	72	283	0.0125
ko00190	oxidative phosphorylation	51	363	0.0145
ko00562	inositol phosphate metabolism	41	147	0.0166
ko00010	glycolysis/gluconeogenesis	34	207	0.0208
ko04380	osteoclast differentiation	63	252	0.0241
ko04060	cytokine-cytokine receptor interaction	70	288	0.0290
ko04640	thyroid hormone signaling pathway	77	381	0.0295
ko04392	fatty acid elongation	8	41	0.0308
ko00020	glycerolipid metabolism	12	108	0.0335

**Table 5:** Osmoregulation-related differentially expressed genes (DEGs) regulated after salinity challenge.

Gene name	Pathway ID	Log2 (Fold Change)
Calcium-transporting ATPase 4 (*ATP2B4*)	K05850	3.637
Na^+^/K^+^-ATPase alpha subunit (*α-NKA*)	K01539	3.248
Potassium-transporting ATPase alpha chain 1 (*ATP4A*)	K01542	4.432
Cation-transporting ATPase 13A3 (*ATP13A3*)	K14951	2.659
Ras GTPase-activating protein (*RasGAP*)	K16848	3.095
GTP-binding protein alpha subunit (*Gq*)	K07902	3.937
Ca^2+^/calmodulin-dependent protein kinase (*CaM*)	K08803	3.150
Adenylate cyclase (*AC*)	K08049	2.095
STE20-like kinase (*SLK*)	K08836	1.285
Serine/threonine-protein kinase WNK1 (*WNK1*)	K08867	2.554
H^+^/Cl^−^ exchange transporter 5 (*CLCN5*)	K05012	3.412
Calcium activated chloride channel (*CaCC*)	K19496	1.904
Potassium channel tetramerization domain containing 10 (*KCTD10*)	K15074	2.173
Potassium channel tetramerization domain containing 13 (*KCTD13*)	K15074	4.269
Potassium channel tetramerization domain containing 20 (KCTD20)	K10482	1.288
C-type lectin (*CRO*)	K06560	4.294
B-cell lymphoma-2 (*Bcl-2*)	K14021	2.705
Heat shock 70 kDa protein (*Hsp70*)	K03283	−3.171
Heat shock 90 kDa protein (*Hsp90*)	K04079	−2.489
IAP repeat-containing protein 6 (*BIRC6*)	K10586	3.212
Programmed cell death 6-interacting protein (*PDCD6*)	K12200	1.561
Protein-tyrosine kinase 6 (*PTK6*)	K05871	1.855

#### Lipid metabolism related pathways

From KEGG pathway analysis of DEGs, fatty acid elongation, fatty-acid degradation, biosynthesis of unsaturated fatty-acids, fatty-acid biosynthesis, these four of enriched pathways related to lipid metabolism are examined. The long-chain polyunsaturated fatty acid level in euryhaline and migratory teleosts raised during salinity acclimation has been reported by many researches^29–31^. Fatty acid desaturases and elongases are the key enzymes in biosynthetic processes from C18 PUFAs to C20-22 LC-PUFAs^32^. In present, Δ6 fatty acid desaturase and elongase 5 in unsaturated fatty-acids and fatty-acid elongation pathways are found significantly up-regulated (4.62 fold and 4.70 fold up-regulated, respectively) in salinity challenge group compared to control group. These results are consistent with the research in euryhaline fish species such as red sea bream^33^, mexican whitefish^34,35^ and Atlantic salmon^36^, which showed that the fatty acid desaturase and elongase expression were significantly up-regulated by ambient salinity stress^37^. A specific physiological function for lipid metabolism related to osmoregulation was confirmed according to these results. As structural components of plasma membrane, the fluidity of cell membranes were enhanced by a larger percentage of LC-PUFAs^38,39^, result in the change of many other physical membrane properties, asn affects the membrane-protein binding^40^. This could be used to explain the raised LC-PUFAs level in euryhaline and migratory teleosts during salinity acclimation.

#### Tight Junction pathway

As known, the tight junction complex plays a significant role in regulating epithelial permeability of vertebrates^41^. With hypersaline concentrations, epithelial morphological changes and intercellular tight junction damage are observed^42^. This observation suggests the significant position of the tight junction pathway in the process of osmoregulation. Claudins, occludin and junctional-adhesion molecule as a group of integral membrane proteins constitute the chordates zonula occludens^43^. Occludin is a broadly expressed tetraspan transmembrane tight junction protein in vertebrate epithelia^44^. In some aquatic vertebrates, It has been reported that the occludin are involved in regulating epithelial permeability^45^, moreover, the large family of claudin isoforms were also proved to relevant to paracellular regulation of hydromineral balance in fish^41,46^. Now, the claudin abundance elevated to 4.42 folds in challenge group compared to that in control group, also, the junctional-adhesion molecule abundance was strongly up-regulated and the fold change of JAM3 was 6.44, which indicated paracellular permeability across diverse epithelia and endothelia of the gills was reduced in salinity challenge for sturgeons.

#### Thyroid hormone signaling pathway

The ability of salinity acclimation not just depend on osmoregulatory capacity but also metabolic reorganization to make related energetic support. Under this background, it’s sensible to find this pathway appears in the top pathway list. There were two iodothyronine deiodinases involve in outer ring deiodination. The type I iodothyronine deiodinas (DIO1) gene was found up-regulated significantly, this may relate to high ORD activities in the salinity challenge gill. Many paper also presented similar express of high ORD activities in the tissues of fish^47–49^. Thyroid hormones play a vital role in lipid, carbohydrate and protein metabolism^50–52^. More importantly, at some segments, Na^+^, K^+^-ATPase activity of the gills and the density of epithelium of cells riched in mitochondria can be significantly raised by thyroid hormones during the environmental salinities acclimation^53,54^. As stated above, thyroid hormone signaling pathway is likely to be critical because its stimulation on basal metabolic rate and energy supply.

### The validation of differently expressional genes by qPCR

**Figure 4:**
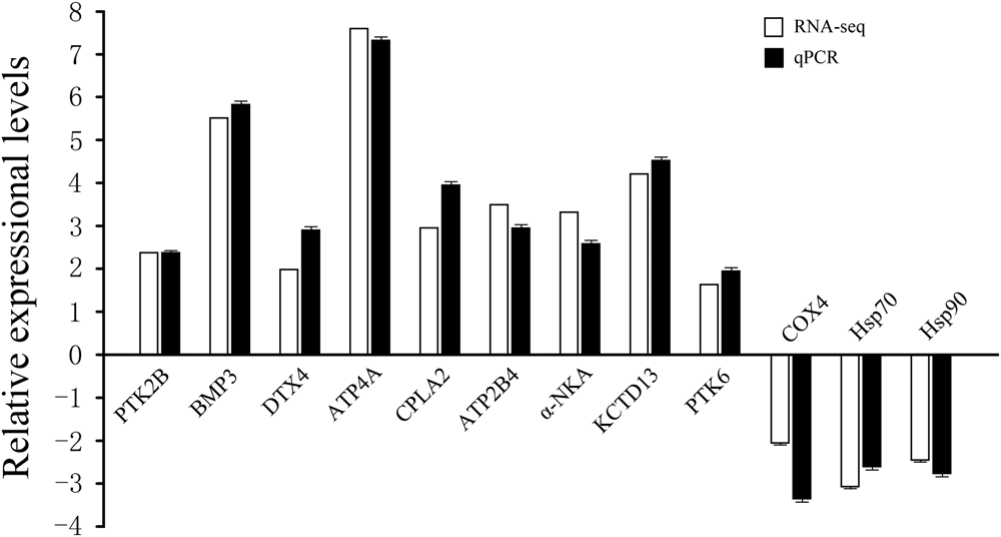
Validations of differently expressed genes in RNA-seq with qPCR. y-axis: the fold change of gene expression, x-axis: the gene name. PTK2B = Protein-tyrosine kinase 2-beta, BMP3 = Bone morphogenetic protein 3, DTX4 = E3 ubiquitin-protein ligase DTX4, COX4 = Cytochrome c oxidase subunit 4, ATP4A = Potassium-transporting ATPase alpha chain 1, CPLA2 = Cytosolic phospholipase A2, ATP2B4 = Calcium-transporting ATPase 4, α-NKA = Na^+^/K^+^-ATPase alpha subunit, KCTD13 = Potassium channel tetramerization domain containing 13, PTK6 = Protein-tyrosine kinase 6, Hsp70 = Heat shock 70 kDa protein and Hsp90 = Heat shock 90 kDa protein.

qPCR was applied in detecting the relative mRNA expression levels of 12 genes from DGE libraries, and there were 9 up-regulated genes and 3 down-regulated genes. The analysis of melting-curve of qPCR illustrated a single product for all genes. Compared fold changes from qPCR with the DGE analysis results, 12 genes showed a consistent direction in both DGE library and qPCR analysis (Fig. 4), proving that the results of RNA-seq (Quantification) was reliable.

## Conclusion

Here, the transcriptome of Siberian sturgeon *A. baeri* response to salinity stress was examined following the application of RNA-seq. The present research described for the first time the effects of salinity on gilled classic osmoregulation-related genes and signaling pathways during acclimation to highly saline environment in sturgeon. Further, a series of other ion transport mechanisms are thus described in the gills hereby, offering a fairly complete picture of the osmoregulatory system of this species when acclimated to high salinity stress. These findings would be useful to develop new aquaculture model in culture industry.

## Data Availability

The datasets generated during and/or analysed during the current study are available from the corresponding author on reasonable request.
